# Metabolomics-based Sleepiness Markers for Risk Prevention and Traffic Safety (ME-SMART): a monocentric, controlled, randomized, crossover trial

**DOI:** 10.1186/s13063-023-07154-x

**Published:** 2023-02-21

**Authors:** Michael Scholz, Stefan Lakaemper, Kristina Keller, Akos Dobay, Andrea Eva Steuer, Hans-Peter Landolt, Thomas Kraemer

**Affiliations:** 1 Department of Forensic Pharmacology and Toxicology, Zurich Institute of Forensic Medicine, University of Zurich, Zurich, Switzerland; 2 Department of Traffic Medicine, Zurich Institute of Forensic Medicine, University of Zurich, Zurich, Switzerland; 3 Forensic Machine Learning Technology Center, University of Zurich, Zurich, Switzerland; 4 Institute of Pharmacology & Toxicology, University of Zurich, Zurich, Switzerland; 5 Sleep & Health Zurich, University of Zurich, Zurich, Switzerland

**Keywords:** Metabolomics, Oral fluid, Biomarker, Sleepiness, Sleep deprivation, Sleep restriction, Drowsy driving, Driving simulation, Fitness to Drive, LC–MS

## Abstract

**Background:**

Too little sleep and the consequences thereof are a heavy burden in modern societies. In contrast to alcohol or illicit drug use, there are no quick roadside or workplace tests for objective biomarkers for sleepiness. We hypothesize that changes in physiological functions (such as sleep–wake regulation) are reflected in changes of endogenous metabolism and should therefore be detectable as a change in metabolic profiles. This study will allow for creating a reliable and objective panel of candidate biomarkers being indicative for sleepiness and its behavioral outcomes.

**Methods:**

This is a monocentric, controlled, randomized, crossover, clinical study to detect potential biomarkers. Each of the anticipated 24 participants will be allocated in randomized order to each of the three study arms (control, sleep restriction, and sleep deprivation). These only differ in the amount of hours slept per night. In the control condition, participants will adhere to a 16/8 h wake/sleep regime. In both sleep restriction and sleep deprivation conditions, participants will accumulate a total sleep deficit of 8 h, achieved by different wake/sleep regimes that simulate real-life scenarios. The primary outcome is changes in the metabolic profile (i.e., metabolome) in oral fluid. Secondary outcome measures will include driving performance, psychomotor vigilance test, d2 Test of Attention, visual attention test, subjective (situational) sleepiness, electroencephalographic changes, behavioral markers of sleepiness, changes in metabolite concentrations in exhaled breath and finger sweat, and correlation of metabolic changes among biological matrices.

**Discussion:**

This is the first trial of its kind that investigates complete metabolic profiles combined with performance monitoring in humans over a multi-day period involving different sleep–wake schedules. Hereby, we aim to establish a candidate biomarker panel being indicative for sleepiness and its behavioral outcomes. To date, there are no robust and easily accessible biomarkers for the detection of sleepiness, even though the vast damage on society is well known. Thus, our findings will be of high value for many related disciplines.

**Trial registration:**

ClinicalTrials.gov Identifier NCT05585515, released on 18.10.2022; Swiss National Clinical Trial Portal SNCTP000005089, registered on 12 August 2022.

## Administrative information

Note: the numbers in curly brackets in this protocol refer to SPIRIT checklist item numbers. The order of the items has been modified to group similar items (see http://www.equator-network.org/reporting-guidelines/spirit-2013-statement-defining-standard-protocol-items-for-clinical-trials/).

**Table:** 

Title {1}	Metabolomics-based Sleepiness Markers for Risk Prevention and Traffic Safety (ME-SMART): a monocentric, controlled, randomized, crossover trial
Trial registration {2a and 2b}.	ClinicalTrials.gov Identifier NCT05585515, SNCTP000005089
Protocol version {3}	Version 1 (14.10.2022)
Funding {4}	Fonds für Verkehrssicherheit FVS, 701.22.01
Author details {5a}	^1^Department of Forensic Pharmacology and Toxicology, Zurich Institute of Forensic Medicine, University of Zurich, Zurich, Switzerland ^2^Department of Traffic Medicine, Zurich Institute of Forensic Medicine, University of Zurich, Zurich, Switzerland ^3^Forensic Machine Learning Technology Center, University of Zurich, Zurich, Switzerland ^4^Institute of Pharmacology & Toxicology, University of Zurich, Zurich, Switzerland ^5^Sleep & Health Zurich, University of Zurich, Zurich, Switzerland *Shared last authorship
Name and contact information for the trial sponsor {5b}	Department of Forensic Pharmacology and Toxicology, Zurich Institute of Forensic Medicine, University of Zurich, Winterthurerstrasse 190/52, 8057 Zurich, Switzerland (Professor Thomas Kraemer: thomas.kraemer@irm.uzh.ch)
Role of sponsor {5c}	This in an academic sponsor-initiated trial. The funder of the study has no role in study design; collection, management, analysis, and interpretation of data; writing of the report; and the decision to submit the report for publication.

## Introduction

### Background and rationale {6a}

Too little sleep and the consequences thereof are a heavy burden in modern societies. Estimating that people sleep on average up to 2 h less over the last decades, sleepiness needs to be considered as a significant societal problem of our modern world [1, 2]. The Swiss Federal Roads Office (FEDRO) states that overtiredness and microsleep are among the top 5 main causes of motorway accidents leading to injuries between 2017 and 2021 [3]. In addition, the Swiss Council for Accident Prevention estimates that drowsy drivers cause 10–20% of all driving accidents [4]. Young males and shift workers seem to be prevalently affected here [5, 6]. The Swiss National Accident Insurance Fund (Suva) estimates that 53,000 occupational accidents per year are sleep-related, producing costs of 283 million Swiss francs [7].

In contrast to alcohol or illicit drug use, there are no quick roadside or workplace tests for objective biomarkers for sleepiness. High inter-individual variability and subjective misinterpretations of sleepiness and sleep propensity provide a challenge to the development of objective sleepiness markers [8–10]. Although numerous different scales to assess sleepiness from different clinical perspectives do exist (for example, Karolinska Sleepiness Scale, Stanford Sleepiness Scale, Epworth Sleepiness Scale, and Johns Scale of Drowsiness), none of them can contain and evaluate all forms of sleepiness at once and therefore no gold standard exists [11]. Hence, objective and quantifiable biomarkers of acute sleepiness are urgently needed, not only for post hoc analysis but also for prevention.

### Objectives {7}

The main objective is built on the central hypothesis of biomarker discovery in metabolomics: Changes in physiological functions (such as sleep–wake regulation) are reflected in changes of endogenous metabolism and should therefore be detectable as change in metabolic profiles. Virtually all aspects of sleep–wake regulation are, at least in part, genetically determined and therefore, must have a molecular substrate. We hypothesize that increasing sleep drive or impaired wakefulness can be assessed by qualitative or quantitative fluctuations of certain metabolites in biological specimens, e.g., by accumulation or decrease of endogenous substances related to sleep propensity. Furthermore, a connection between metabolic profiling and sleepiness-induced performance impairment (especially the ability to drive) has never been drawn before. This study will allow for creating a reliable and objective panel of candidate biomarkers being indicative for sleepiness and its behavioral outcomes.

To avoid the limitations of previous studies, we paid special attention to taking into account specific aspects of human sleep–wake physiology in the study design, as well as the basics and subtleties of biological specimen sampling [12]. Oral fluid has proven its massive potential in metabolomics studies. Being an easily accessible and non-invasive biological fluid suitable for roadside or workplace testing, it overcomes the disadvantage of delay between the time of incident and time of sampling when relying on blood. It is therefore our matrix of choice for the systematic profiling of metabolites across all study blocks. Other alternative specimens such as finger sweat, exhaled breath, and dried blood spots will also be collected. All samples can be analyzed with standard forensic laboratory equipment (i.e., LC–MS).

### Trial design {8}

This is an exploratory, monocentric, controlled, randomized, crossover, clinical study to detect potential biomarkers. Each participant will be allocated in randomized order to each of the three study arms (control, sleep restriction, and sleep deprivation). Due to the nature of the study, blinding is not possible among participants and study personnel. Test assessors will be blinded, however.

## Methods: participants, interventions, and outcomes

### Study setting {9}

This monocentric study will be performed solely on the premises of the University of Zurich, Zurich, Switzerland.

### Eligibility criteria {10}

Previous studies revealed that salivary metabolite profiles are (to a different degree) biased by sex, age, body mass index (BMI), oral hygiene, oral conditions, diet, medication, and lifestyle habits like smoking or alcohol consumption [13, 14]. To control for these and possible sleep–wake-related confounding factors, candidate participants will need to fulfill the following.

Inclusion criteria:

- Male sex
- Age between 20 and 35 years
- Understanding and spoken command of the German language
- Good health condition
- BMI between 18.5–24.9 kg/m^2^
- Habitual average sleep duration between 7 and 9 h/night
- Habitual consumption of 3 or fewer caffeinated beverages/day
- Habitual consumption of 5 or fewer alcoholic beverages/week
- Good sleep quality: Pittsburgh Sleep Quality Index score ≤ 5
- Reasonable oral hygiene (≥ 1 tooth brushing/day)
- Normal or corrected-to-normal vision
- Car driving license holder since at least 2 years (obtained in a country with right-hand traffic) and regular driver (≥ 1 per week)

Exclusion criteria:

- Two or more time zone crossings in the last 3 months
- Habitual napper
- History or presence of neurological disorder, psychiatric disorder, cardiovascular disorder, dental disorder, or any disorder that could pose a risk in participating or that could possibly influence study measurements
- History or presence of a sleep disorder (screening night)
- Use of illicit drugs (urinary drug screening)
- Use of current medication (urinary drug screening) known to influence study measurements
- Extreme chronotype (reduced Morningness-Eveningness-Questionnaire score ≤ 7 or ≥ 22)
- Current smoker
- Habitual use of energy drinks (> 1/week)
- Severe skin allergies or hypersensitivities
- Food allergies
- Hospital stay in the past 6 months
- Shift worker, night worker
- Recent past (last 3 months) or present COVID-19 infection
- Fainting at the sight of blood or needles
- Participation in a clinical study less than 30 days ago or is currently participating in other clinical studies
- Simulator sickness syndrome
- Refusal to sign informed consent

### Who will take informed consent? {26a}

Study personnel obtain written informed consent from potential trial participants prior to any study measure.

### Additional consent provisions for collection and use of participant data and biological specimens {26b}

If not yet covered in this study’s informed consent form, a signed consent must be obtained from every participant in ancillary studies.

## Interventions

### Explanation for the choice of comparators {6b}

Two sleep deficit interventions (sleep deprivation and sleep restriction) were designed to simulate highly prevalent real-life sleep scenarios. Whereas all-night sleep deprivation mimics a night shift work or going out, the sleep restriction regime mimes a typical workweek schedule with an equal total sleep loss than all-night sleep deprivation. Cumulative effects of sleep restriction leading to performance impairment and increased crash risk have been described before**,** but their impact on metabolic outcomes is unknown [10, 15, 16].

While a constant routine protocol (e.g., permanent dim light conditions, strictly controlled body posture) is necessary to avoid masking in circadian studies, a slightly reduced level of control is deemed sufficient in the present study focused on sleep–wake rather than circadian perturbation.

### Intervention description {11a}

**Fig. 1:**
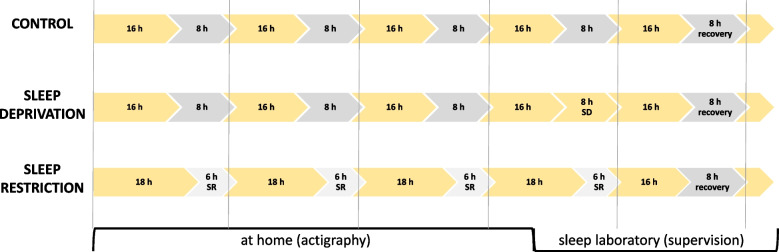
Overview of sleep regime in different study arms (control, sleep deprivation, sleep restriction). Yellow colors indicate time awake, gray colors bed times, vertical lines a period of 24 h. For further explanations, see the text. Abbreviations: SD, sleep deprivation; SR, sleep restriction

Three conditions that only differ in the amount of hours slept per night will be studied (see Fig. 1).

In the control (C) condition, participants will adhere to a 16/8-h wake/sleep regime for 3 consecutive days at home and for the following 2 days in the sleep laboratory under the supervision of study personnel.

In the sleep deprivation (SD) condition, participants will follow a 16/8-h wake/sleep regime for 3 consecutive days at home and one night of supervised sleep deprivation (24/0 h) in the sleep laboratory, thus generating a total sleep deficit of 8 h compared to the control condition. The following night (recovery night) offers 8 h of supervised sleep.

In the sleep restriction (SR) condition, participants will follow an 18/6-h wake/sleep regime for 3 consecutive days at home and 1 day in a sleep laboratory under the supervision of study personnel, thus also accumulating a total sleep deficit of 8 h compared to the control condition. The following night (recovery night) offers 8 h of supervised sleep.

### Criteria for discontinuing or modifying allocated interventions {11b}

Discontinuation or modification of allocated interventions is not planned. Discontinuation is possible in case of adverse events or if participants do not adhere to protocol requirements. In addition, if organizational or the participant’s situation require, a switch between pre-scheduled control and sleep deprivation conditions may be allocated at the participant’s arrival in the sleep laboratory. These two conditions only differ in the amount of scheduled sleep in the subsequent night. The modification in the allocated conditions may permit the efficient usage of resources, and thus reduce the total trial duration and facilitate intra-individual comparability of study specimens if collected in shorter time intervals.

### Strategies to improve adherence to interventions {11c}

Participants’ adherence to sleep regimes will be monitored by the use of wrist actigraphy and sleep–wake diaries. At the time of arrival at the sleep laboratory, a urine sample will be collected and tested for recent drug use, as well as a breath test to detect recent alcohol consumption. Once at the sleep laboratory, participants will be under constant supervision of study personnel.

### Relevant concomitant care permitted or prohibited during the trial {11d}

Medical care will be available throughout the study. If participants need to seek medical care during the trial period or start taking medication, the principal investigator will decide whether the participant will have to be excluded or whether the study can be completed after completed recovery.

### Provisions for post-trial care {30}

A general follow-up appointment is not foreseen. Participants who withdraw from the study due to adverse events will undergo a follow-up check up to 30 days after exclusion. Compensation to those who suffer harm from trial participation will be paid according to policy conditions concluded by the University of Zurich and the respective insurance company.

### Outcomes {12}

The primary outcome of this trial is changes in the metabolic profile (i.e., metabolome) in oral fluid. Investigators will collect samples from participants for quantification of all detectable metabolites after arrival at the study site (6 pm, baseline), repeatedly during scheduled wakefulness (8 am, 12 pm, 4 pm, 7 pm, 11 pm), and in the morning after recovery night (8 am). They will analyze which metabolite concentration values undergo significant changes during sleep deficit conditions in comparison to the control condition and also show the effects of recovery sleep. These will serve as candidate biomarkers.

Secondary outcome measures will include:

i. Driving performance: Investigators will gather driving simulation results via Standardized Application for Fitness to Drive Evaluations (S.A.F.E.) [17–19], and analyze their changes after sleep deficit in comparison to the control condition. Driving simulation test is scheduled once per study arm, in the morning after the experimental night (10 am).
ii. Psychomotor vigilance test (PVT) [20]: The PVT is a gold standard reaction time test to assess vigilance and sustained attention. It will be performed after arrival at the study site (6 pm, baseline), repeatedly during scheduled wakefulness (8 am, 12 pm, 4 pm, 7 pm, 11 pm), and in the morning after recovery night (8 am).
iii. d2 Test of Attention [21, 22]: The d2 Test of Attention is a paper and pencil test to assess selective and sustained attention and visual scanning speed. It is scheduled after arrival at the study site (6 pm, baseline), repeatedly during scheduled wakefulness (8 am, 12 pm, 4 pm, 7 pm, 11 pm), and in the morning after recovery night (8 am).
iv. Visual attention test [23]: The visual attention test is a virtual reality glasses test to assess visual skills in a complex visual environment. It will be conducted after arrival at the study site (8 pm, baseline), repeatedly during scheduled wakefulness (10 am, 2 pm, 6 pm, 8 pm), and in the morning after recovery night (8 am).
v. Subjective situational sleepiness: Participants will complete the Karolinska Sleepiness Scale (KSS) [24] questionnaire after arrival at the study site (6 pm, baseline), repeatedly during scheduled wakefulness (8 am, 12 pm, 4 pm, 7 pm, 11 pm), and in the morning after recovery night (8 am).
vi. Subjective sleepiness: Participants will complete the Stanford Sleepiness Scale (SSS) [25] questionnaire after arrival at the study site (6 pm, baseline), repeatedly during scheduled wakefulness (8 am, 12 pm, 4 pm, 7 pm, 11 pm), and in the morning after recovery night (8 am).
vii. Electroencephalographic (EEG) changes: Investigators will analyze changes in sleep and wake EEG patterns of participants by calculating sleep scores according to the American Academy of Sleep Medicine (AASM) scoring manual [26]. EEG recordings will be performed during scheduled sleep, driving simulation test (10 am), and at two time points during scheduled wakefulness (12 pm, 7 pm).
viii. Behavioral markers of drowsy driving: Investigators will examine participants immediately after the driving simulation test for behavioral abnormalities concerning orientation, coordination, speech, mood, appearance, reaction, and pupillary light reflex.
ix. Changes in metabolite concentrations in exhaled breath: Investigators will collect exhaled breath samples from participants for quantification of all detectable metabolites after arrival at the study site (8 pm, baseline), repeatedly during scheduled wakefulness (10 am, 2 pm, 6 pm, 8 pm, 11 pm), and in the morning after recovery night (8 am). They will analyze which metabolite concentration values undergo significant changes during sleep deficit conditions sessions in comparison to the control condition and also show the effects of recovery sleep. These will serve as secondary candidate biomarkers.
x. Changes in metabolite concentrations in finger sweat: Investigators will collect finger sweat samples from participants for quantification of all detectable metabolites after arrival at the study site (8 pm, baseline), repeatedly during scheduled wakefulness (10 am, 2 pm, 6 pm, 8 pm, 11 pm), and in the morning after recovery night (8 am). They will analyze which metabolite concentration values undergo significant changes during sleep deficit conditions sessions in comparison to the control condition and also show the effects of recovery sleep. These will serve as secondary candidate biomarkers.
xi. Correlation of metabolic changes between blood and non-invasive specimens: Investigators will compare metabolite concentrations in non-invasive matrices (oral fluid, finger sweat, exhaled breath, and dried blood spots) and compare those with a blood sample. The collection of specimens for this outcome is scheduled immediately after the driving simulation test in each study arm.

### Participant timeline {13}

**Fig. 2:**
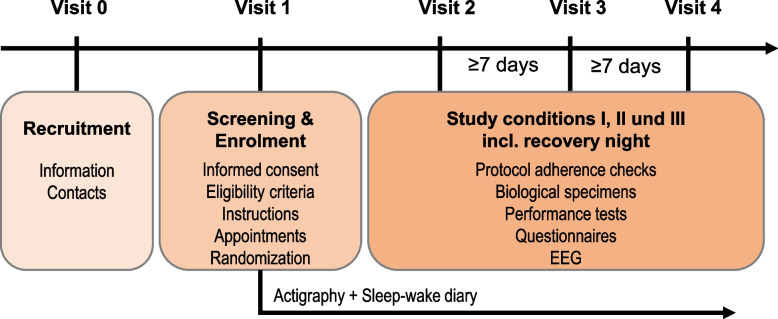
Participant timeline. Washout time in between study session visits is at least 7 days

The time schedule of recruitment, screening and enrolment, and study session visits is shown in Fig. 2.

### Sample size {14}

For sample size estimation and power calculation, we analyzed untouched saliva samples of a previous sleep deprivation study, in which participants underwent 40 h of prolonged wakefulness and a control condition including 8 h of sleep in random, crossover fashion [27]. We detected more than 50 metabolic features showing a significant (*p* < 0.05, paired *t*-test) difference between the sleep deprivation and control conditions (unpublished observation). Utilizing the results of preliminary analysis for a power calculation (*α* = 5%, *β* = 20, thus the power of 80%), a sample size of 17 participants will be sufficient to detect significantly different metabolite quantities (*p* < 0.05). As the presented study setting offers several improvements for meeting the defined outcome goals, a smaller sample size may be appropriate. Nevertheless, taking into account a few possible dropouts of participants (e.g., risk of driving simulator sickness) and organizational aspects (number of available beds in sleep research laboratory per session is four, adequate supervision, etc.), and ultimately assuring ourselves to perform a sufficiently powered study, we aim to recruit 24 participants.

For comparison, relevant completed trials with comparable protocols had sample sizes between 10 and 16 (10 male, 12 male, 12 female, and 16 (eight men)) [28–31].

### Recruitment {15}

Recruitment of volunteers will be accomplished by a three-step escalation plan. Firstly, premises, networks, and extensive media channels of the University of Zurich and local schools will be used for advertisement of the trial. As a second means, public spaces and displays in Zurich will inform about the open recruitment status. Lastly, trial information can be spread via partner universities and public media channels over the whole Switzerland and Southern Germany. The recruitment period is scheduled from November 2022 until June 2023.

## Assignment of interventions: allocation

### Sequence generation {16a}

The order of interventions will be defined by random envelope picking of participants after successful passing of screening tests. Given three study arms (C, SD, SR), there are six different possible orders of interventions. Each order will be performed four times in an equal ratio. The first 18 participants will pick their respective assignment envelope from box A which contains all possible orders in triplicates (3:3:3:3:3:3), thus guaranteeing an equal allocation ratio until the minimum sample size is reached. The remaining participants will draw from box B, where each order is contained once (1:1:1:1:1:1).

### Concealment mechanism {16b}

Concealment is ensured by paper assignments kept in sealed, opaque envelopes.

### Implementation {16c}

The sponsor of the study will prepare the assignment envelopes. As he is not part of enrolling and assigning procedures, this allows for allocation concealment.

## Assignment of interventions: blinding

### Who will be blinded {17a}

Due to the nature of the study interventions, neither participants nor study personnel can be blinded. To ensure an unbiased rating of participants, outcome assessors of respective performance tests will be blinded.

### Procedure for unblinding if needed {17b}

N/a. Since participants and study personnel cannot be blinded, there is no unblinding. Outcome assessors must be kept blinded even after tests to prevent biased ratings of following participants.

## Data collection and management

### Plans for assessment and collection of outcomes {18a}

All data and biological specimens will be collected by trained study personnel. Standard operating procedures (SOP) for each study activity ensure consistent and high data quality. Participants will familiarize themselves with the equipment and performance tests (especially the driving simulator) during screening to prevent learning effects during the study sessions.

For the primary outcome, unstimulated oral fluid will be collected using neutral Salivette® (Sarstedt, Sevelen, Germany) sampling devices. Samples will be stored at − 80 °C until trial conclusion and then extracted and analyzed in an untargeted metabolomics approach by ultra-performance liquid chromatography coupled to high-resolution mass spectrometry (UPLC-HRMS) equipment.

For the secondary outcome measures, different procedures for data collection and assessment will be applied:

i. Driving simulation will take place in a custom-modified full-chassis BMW i3 car, in which actuators replace shock absorbers to convey motion cues, such as road bumps, acceleration and braking. Placed in a 270-degree-projection and 360-degree-visibility setting, participants will drive through a fully immersive simulated environment provided by the driving simulation software (SILAB, “Szenariopaket Driver Fitness and Ability” Version 7.0; Würzburg Institute for Traffic Sciences GmbH, Veitshöchheim, Germany), offering a row of validated scenario modules. SILAB simultaneously records vehicle and driving parameters to evaluate, among others, standard deviation of the lateral position (SDLP). Standard driving errors are recorded automatically via Standardized Application for Fitness to Drive Evaluations (S.A.F.E.) software. Additionally, S.A.F.E. allows including observed qualified errors and scoring the driving performance (Fitness to Drive, FtD) [17–19]. Participants perform a two-part customized driving task. An approximately 12-min-long, highly monotonous car following task in a nighttime scenario, including a sound-based vigilance task [18], followed by an approximately 8-min-long, early-morning rural road scenario.
ii. The PVT will be performed on laptops and its results will be calculated according to the validated protocol [20].
iii. The d2 Test of Attention will be performed on paper and its results will be calculated according to a standardized manual [21, 22].
iv. Visual attention test will be performed using virtual reality glasses according to standardized procedure and assessment [23].
v. Paper-based Karolinska Sleepiness Scale (Version A) questionnaires will be provided and assessed according to the validated procedure [24].
vi. Validated Stanford Sleepiness Scale questionnaires will be completed on laptops and follow a simple seven-scale choice [25].
vii. The EEG data, complemented by the polysomnographic (PSG) recording of electrooculogram (EOG), electromyogram (EMG), and electrocardiogram (ECG), will be collected with SIENNA® Ultimate amplifier systems (EMS Biomedical, Korneuburg, Austria) and analyzed with dedicated software for the visual scoring of sleep variables (e.g., alphatrace NeuroSpeed-PSG, alpha trace medical systems, Vienna, Austria). During the driving simulation, EEG data will be recorded using a mobile Philips ALICE 6 device (Philips AG Sleep and Respiratory Care, Zofingen, Switzerland). According to standard international criteria, sleep stages will be scored for each night in the laboratory, and validated sleep variables reflecting sleep quality (e.g., total sleep time, sleep latency, length of non-rapid-eye-movement (NREM) and rapid-eye-movement (REM) sleep stages, waking time after sleep onset, sleep efficiency) will be derived and compared between the conditions [26]. Labmade software will be used for the quantitative analysis of the EEG.
viii. Blinded and pre-instructed assessors will collect the behavioral abnormalities of participants after the driving simulation test on paper. They will rate orientation, coordination, speech, mood, appearance, reaction, and eye reactions to light according to standardized options.
ix. Unstimulated finger sweat will be collected using self-punched sampling units made of filter paper. Samples will be handled and analyzed analogously to oral fluid samples.
x. Exhaled breath samples will be collected in specialized devices from SensAbues AB (Stockholm, Sweden) and BreathExplor® (Munkplast AB, Uppsala, Sweden). Dried blood spots will be collected on volumetric cards (Capitainer, Solna, Sweden). All samples will be handled and analyzed analogously to oral fluid samples.

### Plans to promote participant retention and complete follow-up {18b}

Appointments for study interventions will be scheduled with the participants directly after successful completion of screening, and a personal reminder will be sent in advance. Financial compensation will be paid per complete study arm and increases to promote retention. Participants will also be offered presentation of study results after completion, if interested.

For participants deviating from intervention protocols, non-adherence will be recorded in the CRF. If deviations concern sleep regime, all outcome data will be excluded from further analysis.

### Data management {19}

SOP instruct the entire study personnel on how to make data entries to eliminate differences. Protocol procedure data will be collected in a pseudonymized paper CRF where all entries have to be signed and dated by study personnel. Raw data of performance tests and biological specimen analyses will be stored digitally on local computers with access rights restricted to study personnel only. Two independent study employees will digitalize the results of paper-based performance tests and questionnaires after every session. Daily backups of local IT infrastructure secure digital data. Additional backups of digital raw data on password-secured and encrypted external hard drives are automatically run.

All original digital data and paper records, such as CRF, consent forms, and relevant correspondence, will be archived at the sponsor’s institute for a minimum of 10 years after trial completion, according to local regulations.

### Confidentiality {27}

Participants will be allocated an individual ID number after providing written informed consent. This allocation will be kept secure in a password-protected and access-restricted identification log file. Copies of written informed consent forms will be stored separately from study records in restricted area. All other documents, files, labels, and specimens will only contain participants’ ID numbers and are therefore pseudonymized. During the trial period in the sleep laboratory, participants will wear their individual ID number visible on clothing.

Suspension of confidentiality can only be initiated with written permission of the participant or for the purpose of monitoring and audits by entitled authorities.

### Plans for collection, laboratory evaluation, and storage of biological specimens for genetic or molecular analysis in this trial/future use {33}

The coded biological specimens will be stored refrigerated for a maximum of 10 years after trial completion at the Zurich Institute of Forensic Medicine, University of Zurich, Switzerland, and will be destroyed thereafter. Decryption is not foreseen, except by personal request and written permission of respective participant.

## Statistical methods

### Statistical methods for primary and secondary outcomes {20a}

Based on our study design, statistical analysis will be performed to detect both intra- and inter-individual (i.e., individual and group) differences. All the analyses will be carried out in reference to hours of wakefulness (i.e., clock time), and also in reference to the internal body time (IBT) of the participants which is determined by the dim light melatonin onset (DLMO) from oral fluid samples.

The primary outcome of the trial is the changes detected in the metabolome. Concentration values of metabolic features will be used to generate feature time series. As a first characterization, the rhythmicity of features will be tested by cosinor analysis. Other regression models such as linear, exponential, or logarithmic functions will be applied for fitting time series data in order to simplify complex patterns. To extract relevant features from the time series, different statistical approaches will be carried out. Firstly, we will investigate how group-level differences vary when applying generalized linear mixed models and — if group-level differences exist — conduct multiple comparison tests. After applying the corrections for multiple testing (Benjamini–Hochberg procedure, i.e., false discovery rate), adjusted p-values will be considered significant when below 0.05. Significant features will then be shortlisted. Secondly, each time series will be checked for the occurrence of regular patterns (e.g., linear increase/decrease, exponential, etc.) in relation to hours of wakefulness. Again, each feature candidate will be added to the shortlist. Lastly, a classification task will be carried out to isolate those features leading to a separation between the study arms. The result of the primary outcome will be a list of candidate biomarkers.

For secondary outcomes, differences between study groups will be reported. For variables that are continuous (i.e., outcome of PVT, d2-task, driving simulator parameters, EEG data) the mean and the median will be computed accordingly. For variables that are either binary or ordinal (i.e., results of the behavioral impairment test, visual attention test, sleepiness scales, expert decision on driving ability), the relative frequencies, proportions, and standard errors for each group will be reported.

Regarding the metabolic correlation between blood and non-invasive specimens as a secondary outcome, a regression analysis will be performed between metabolite concentration values in blood and in other sampled matrices.

As a last step, causality between all outcomes will be tested by cross-correlation analyses.

### Interim analyses {21b}

N/a. Interim analyses are not planned. This is a low-risk trial; therefore, no stopping guidelines are applied.

### Methods for additional analyses (e.g., subgroup analyses) {20b}

Given the study design, subgroup analyses will be performed as described above. Adjusted analyses are not planned but can be justified if incomplete datasets impact the balance between study conditions.

### Methods in analysis to handle protocol non-adherence and any statistical methods to handle missing data {20c}

Missing datasets due to participants’ withdrawal from the trial will not be imputed. However, if two of the three study datasets are complete (especially if the control condition is available), these can still be used for two-sided study arm comparisons.

Missing data in metabolic feature tables are common. Typical imputation strategies (e.g., gap filling) in the field of metabolomics are already incorporated in raw data processing software. Therefore, processing parameters will be reported in detail.

### Plans to give access to the full protocol, participant-level data and statistical code {31c}

Full protocol and raw data will be made available upon reasonable collaborative request. Statistical code will be made publicly available on the GitHub repository after publishing of results.

## Oversight and monitoring

### Composition of the coordinating center and trial steering committee {5d}

The roles and responsibilities of stakeholders are clearly defined by Good Clinical Practice (GCP) regulations. Meetings of the sponsor, investigator, site manager, and study personnel for progress evaluation are scheduled monthly. In case of adverse events, non-adherence, or other occurring problems, an urgent meeting will be arranged within three days. Sponsor’s PhD student will organize communication to all parties and conduct trial management.

### Composition of the data monitoring committee, its role and reporting structure {21a}

N/a. There is no need for a data management committee as there are no interim analyses. Data analysis will take place after trial completion under the supervision of a statistician independent of the sponsor or funder.

### Adverse event reporting and harms {22}

The sleep interventions (SD and SR) mimic common real-world scenarios. The trial was thus categorized as a low-risk trial by the independent ethics review board. Therefore, we do not expect adverse events to happen. Collection, assessment, reporting, and management of adverse events and other unintended effects of trial interventions or trial conduct will be realized according to local ethics regulations, however. Furthermore, providing a yearly safety report is mandatory.

### Frequency and plans for auditing trial conduct {23}

Monitoring is carried out in accordance with directives of the Swiss Association of Research Ethics Committees (swissethics) and Swiss laws and enactments on human research. An independent and experienced trial monitor has been appointed and approved by the local ethics commission. He will verify study conduct according to the protocol and have insight in all study documents and data under a confidentiality agreement. His monitoring report will be sent to the local ethics committee without prior insight of study stakeholders. Furthermore, the local ethics committee is authorized to audit at any time without prior notification.

### Plans for communicating important protocol amendments to relevant parties (e.g. trial participants, ethical committees) {25}

Major amendments to the protocol need the approval of the local ethics committee before they can be implemented. This especially concerns modifications that may affect the safety or risk exposure of trial participants. Upon approval, necessary protocol amendments will be communicated to all parties, including the participants. Minor changes such as organizational aspects of the study conduct do not need ethical committee approval but will be listed in the yearly safety report.

All study documents are version controlled and follow track change guidelines.

### Dissemination plans {31a}

The results of this study will be published in peer-reviewed scientific journals, regardless of the direction of outcomes. Digital copies will be sent to trial participants, if they expressed their interest. Findings will also be presented on scientific conferences and meetings. A final project report will be sent to the funder.

## Discussion

To the best of our knowledge, this is the first trial of its kind that investigates complete metabolic profiles combined with performance monitoring in humans over a multi-day period involving different sleep–wake schedules (control, sleep deprivation, and sleep restriction conditions in the same participants). Hereby, we aim to establish a candidate biomarker panel being indicative for sleepiness and its behavioral outcomes. For that purpose, established validated performance tests**,** as well as actual driving ability in a highly sophisticated driving simulator will be assessed. Driving simulation will be tested in the early morning hours, which is the peak time of sleep-related car crashes [5, 32]. Thus, by mimicking real-world scenarios, we believe that the generalization of our findings will be well justified. Nevertheless, our intended candidate biomarker panel will have to be verified by routine case samples to be tested for ruggedness towards confounding factors. Since the study only includes men, it remains to be seen whether the biomarker panel also works for women. We decided to recruit only male volunteers due to the strong menstrual cycle’s implications on the female metabolome [30]. However, as sleep–wake regulation represents a fundamental structure not just in humans, we assume that robust markers should exist and can be detected by our study. Taking into account that young males represent the most vulnerable target group of microsleep-related car accidents (compare {6a}), this does not necessarily mean a limitation of study results. In this trial, we chose to apply strict inclusion and exclusion criteria as well as tight control and supervision. In doing so, we hypothesize detecting relevant markers in less than 20 participants. Although the physiological principles underpinning sleep–wake regulation are generalizable, the current findings will be applicable only to healthy young men who form the highest-risk group for sleepiness-induced road traffic accidents. We are convinced that they will expand our knowledge of candidate biomarker panels of sleep–wake regulation in humans. Nevertheless, future studies in larger samples will have to examine whether the findings can be generalized to the general population and to an uncontrolled environment.

Since there are no easily detectable markers for different sleep phenotypes (i.e., chronotypes) yet, we expect that some metabolites will show good applicability in a certain (phenotype) group of subjects but not necessarily outside this given group. To exclude further confounding factors, only standardized, isocaloric meals and non-stimulating drinks will be offered and consumed at the same clock times. Furthermore, equal toothbrushes and additive-free toothpaste will guarantee unbiased oral hygiene comparability for salivary metabolomics analyses. Temperature and light emission will be controlled and match day and night conditions.

To date, there are no robust and easily accessible biomarkers for the detection of sleepiness, even though the vast damage on society (health issues, accidents, etc.) is well known. Thus, our findings will be of high value for many related disciplines.

## Trial status

Protocol version 1 (submitted 14.10.2022). Recruitment is anticipated to begin in late October 2022 and will end as soon as 24 participants have successfully completed the trial or in June 2023, whichever comes soonest.

## Data Availability

Sponsor, sponsor’s PhD student, and principal investigator will have access to the final trial dataset. For analysis and publication, necessary data will be made available to the involved study collaborator for the respective study question. Any data required to support the protocol can be supplied on request.

## Abbreviations

AASM: American Academy of Sleep Medicine
BMI: Body mass index
C: Control
CRF: Case report form
CTC: Clinical Trials Center
DLMO: Dim light melatonin onset
ECG: Electrocardiogram
EEG: Electroencephalography
EMG: Electromyogram
EOG: Electrooculogram
FtD: Fitness to Drive
GCP: Good clinical practice
KSS: Karolinska sleepiness scale
LC–MS: Liquid chromatography with mass spectrometry
NREM: Non-rapid-eye-movement
PSG: Polysomnography
PVT: Psychomotor vigilance test
REM: Rapid-eye-movement
S.A.F.E: Standardized Application for Fitness to Drive Evaluations
SD: Sleep deprivation
SDLP: Standard deviation of the lateral position
SOP: Standard operating procedures
SR: Sleep restriction
SSS: Stanford sleepiness scale
Suva: Schweizerische Unfallversicherungsanstalt (German for Swiss National Accident Insurance Fund)
UPLC-HRMS: Ultra-performance liquid chromatography with high-resolution mass spectrometry
USZ: University Hospital Zurich
